# Systematic video analysis of shoulder dislocations in professional male football (soccer): Injury mechanisms, situational and kinematic patterns

**DOI:** 10.1002/jeo2.70121

**Published:** 2024-12-19

**Authors:** Kristian Nikolaus Schneider, Tim Schachtrup, Georg Gosheger, Mirkka Lynn Hiort, Blanca Julie Degener, Theodoros Zafeiris, Christoph Theil

**Affiliations:** ^1^ Department of Orthopaedics and Tumor Orthopaedics University Hospital of Münster Münster Germany

**Keywords:** acute shoulder injuries, contact sports injuries, football injury patterns, upper extremity injuries

## Abstract

**Purpose:**

Shoulder dislocations in professional football are severe injuries with an increasing incidence and considerable layoff times. Unlike other football injuries, the mechanisms leading to shoulder dislocations are not well understood, which limits the development of targeted preventive measures. Thus, the aims of this study were: (1) to analyse injury mechanisms of shoulder dislocations in professional football, (2) to evaluate situational and (3) to assess kinematic patterns by performing a systematic video analysis.

**Methods:**

The study included all shoulder dislocations occurring in official matches within Germany's top two professional male football (soccer) leagues (1. and 2. Bundesliga) from the 2012/2013 to the 2022/2023 seasons. A systematic video analysis was performed using the official Bundesliga video database. Two independent reviewers assessed injury‐related, situational and kinematic parameters.

**Results:**

A total of 37 shoulder dislocations in 36 players (mean age 25 years) were analysed. Two primary injury mechanisms were identified: Type 1 mechanisms, caused by direct contact to the upper extremity (*n* = 14), and Type 2 mechanisms, caused by catching a fall (*n* = 22). Only one case did not fit these categories. Median layoff times were 94 days for Type 1 and 56 days for Type 2, but this difference was not statistically significant. Statistically significant differences between the two types were found in player action (*p* < 0.001) and type of contact (*p* = 0.005), while factors like player's speed, movement direction, football‐specific actions, ball possession and pitch location showed no statistically significant differences. On‐field treatment methods varied, but there were no statistically significant differences in repositioning attempts or reduction techniques (n.s.). Trunk position, shoulder joint version, shoulder elevation, and rotation, as well as elbow and wrist joint positions at the moment of dislocation, were similar between the two types (n.s.).

**Conclusion:**

Shoulder dislocations in professional football typically occur through direct contact or catching a fall, indicating a potential role for specific preventive measures.

**Level of Evidence:**

Level III.

**Abbreviations**
IQR: interquartile range
n.s.: not significant

## INTRODUCTION

In professional male football within the German Bundesliga, shoulder dislocations currently occur at a rate of approximately 9 per 1000 matches played [26]. These incidents regularly lead to notable layoff times and while the ideal treatment is the subject of ongoing debate, repetitive instability events regularly require surgical intervention [7, 13, 15, 18, 26, 29, 33]. Despite the injury's severity, the underlying injury mechanisms remain poorly understood, partly due to the low incidence compared to other typical injuries in professional football. Notably, existing preventive measures are primarily designed for goalkeepers, despite more than 90% of shoulder dislocations occurring in field players [18, 26]. Previously, systematic video analyses have played a crucial role in enhancing comprehension, treatment, and prevention of common football‐related injuries such as anterior cruciate ligament tears, hamstring, and quadriceps injuries, as well as Achilles tendon tears [2, 6, 8, 9, 11, 34, 35]. Consequently, this systematic video analysis of first‐time in‐match shoulder dislocations in professional male football (1. and 2. Bundesliga) aims to (1) identify the causative injury mechanisms, or the specific physical events and forces that directly result in injury, and to (2) analyse situational patterns, including contextual factors such as player actions, ball possession, and match conditions leading up to the injury. This helps to assemble the external conditions associated with these events. Furthermore, (3) identifying kinematic patterns—the precise body movements and joint positions at the moment of injury—will aid in understanding the biomechanics that heighten susceptibility to shoulder dislocations. Together, these insights are essential for developing targeted preventive strategies and improving clinical and on‐pitch management practices.

## MATERIAL AND METHODS

The study was approved by the local ethics committee (reference number: 2023‐215‐f‐S) and performed in accordance with the Declaration of Helsinki.

Systematic video analysis of all shoulder dislocations within the top two male professional football (soccer) leagues in Germany (1. and 2. Bundesliga) during the seasons 2012/2013 to 2022/2023 as a follow‐up study of a previous epidemiological analysis [26]. During this study period, a total of 6732 matches were included and shoulder dislocations as well as medical treatment and respective lay‐off times were identified through a media analysis previously described and validated [17, 26]. Matches from the three Bundesliga were excluded due to insufficient video availability. Match videos were obtained from the official Bundesliga video base (Sportcast GmbH) and usually provided up to six different camera angles. Videos were evaluated by two independent reviewers following a systematic checklist based on previous systematic video analyses in professional football with specific additions for the assessment of shoulder dislocations [14]. Following an independent individual evaluation, consensus was found in divergent cases.

### General information

General information regarding the player's age, minute and position played by the player, season, match day and playing venue (home/away) were extracted from the video footage and the official Bundesliga database (www.bundesliga.de, Deutsche Fußball Liga GmbH).

### Injury mechanisms

Injury mechanisms were identified by thoroughly examining the entire sequence of play leading up to each shoulder dislocation, utilizing footage from all available camera perspectives. For greater accuracy, an additional slow‐motion analysis was employed to capture precise movement details. Trunk rotation and joint angles were measured specifically at the moment of shoulder dislocation by analysing the athlete's posture and orientation in relation to anatomical landmarks. This involved tracking markers or reference points on the shoulder, arm and torso to calculate the degree of rotation and joint positioning during the incident. These metrics were quantified by assessing the angular displacement between the shoulder joint, elbow and torso alignment, ensuring consistency across cases. The identified mechanisms were then further explored in a sub‐group analysis to determine variations in injury patterns among different mechanisms of shoulder dislocation.

### Situational patterns

Situational patterns evaluated included the football‐specific situation, ball possessing team, location of the injury on the pitch, on‐pitch treatment, as well as the decision‐making of the referee. The player's speed was rated as *fast* when the affected player was sprinting, *medium* when the affected player was jogging and *slow* when the player was walking or standing.

### Kinematic patterns

The kinematic patterns obtained included the rotation of the trunk and the position of the shoulder joint, elbow joint and wrist joint.

### Statistical analysis

Statistical analyses were performed using SPSS 25.0 (IBM Corp.). Categorical variables were compared using cross tables and the chi‐squared test. The data distribution was determined with the Kolmogorov–Smirnov test. Means and ranges are presented for parametric data, and medians and 25%–75% interquartile range (IQR) are presented for non‐parametric data. Non‐parametric analyses were performed with the Mann–Whitney *U* test, and parametric analyses were performed with Student's *t* test. All *p* values were two‐sided and a *p* value of less than 0.05 was considered statistically significant.

## RESULTS

From the 2012/2013 to the 2022/2023 seasons, there were a total of 37 shoulder dislocations among 36 players in the 1. and 2. German Bundesliga leading to an incidence of 5.5 first‐time shoulder dislocations per 1000 Bundesliga matches played. The players' mean age was 25 years (range, 18–33) and the mean minute played by the player up until the dislocation event was 35 (range, 4–91). Overall, the median layoff time for all players was 86 days (IQR, 23–108): 19 days (IQR, 9–31) in players who underwent conservative treatment and 100 days (IQR, 87–131; *p* < 0.001) in players who underwent surgical treatment.

The mean match day of shoulder dislocations was the 18th match day (range, 2–33), with a nearly equal distribution over the first and second half of the season which consists of 34 match days. In the first half, there were 18 (49%) shoulder dislocations, and in the second half, there were 19 (51%). Additionally, 20 (54%) dislocations occurred on the home ground, while 17 (46%) occurred in away matches.

### Injury mechanisms

**Figure 1:**
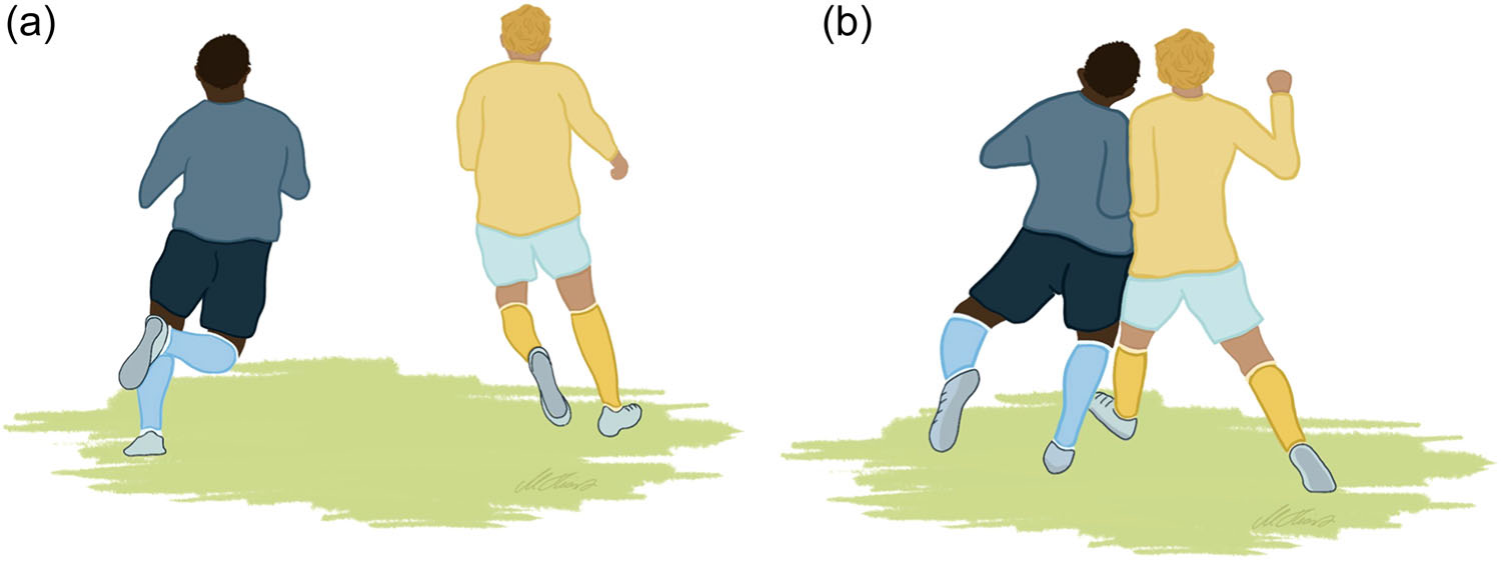
(a and b) Shoulder dislocation following a direct contact (Type 1).

**Figure 2:**
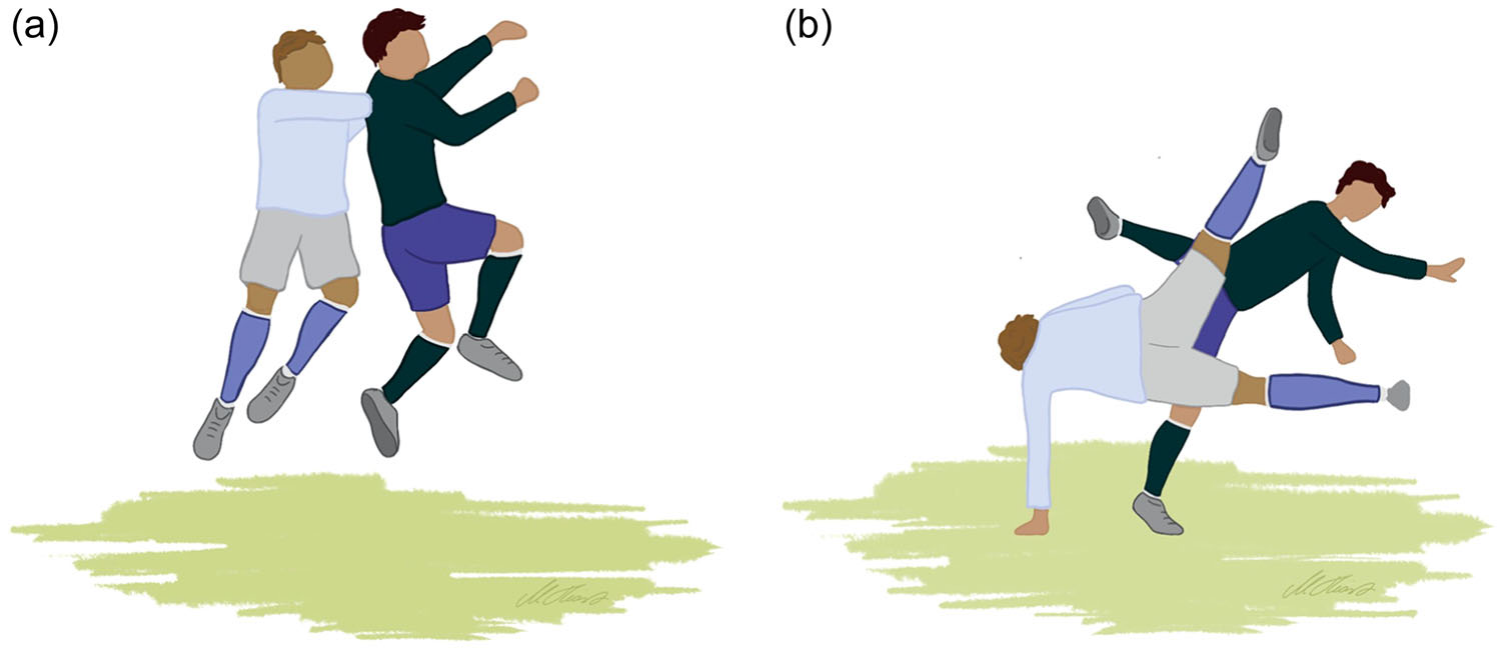
(a and b) Shoulder dislocation following catching a fall (Type 2).

**Figure 3:**
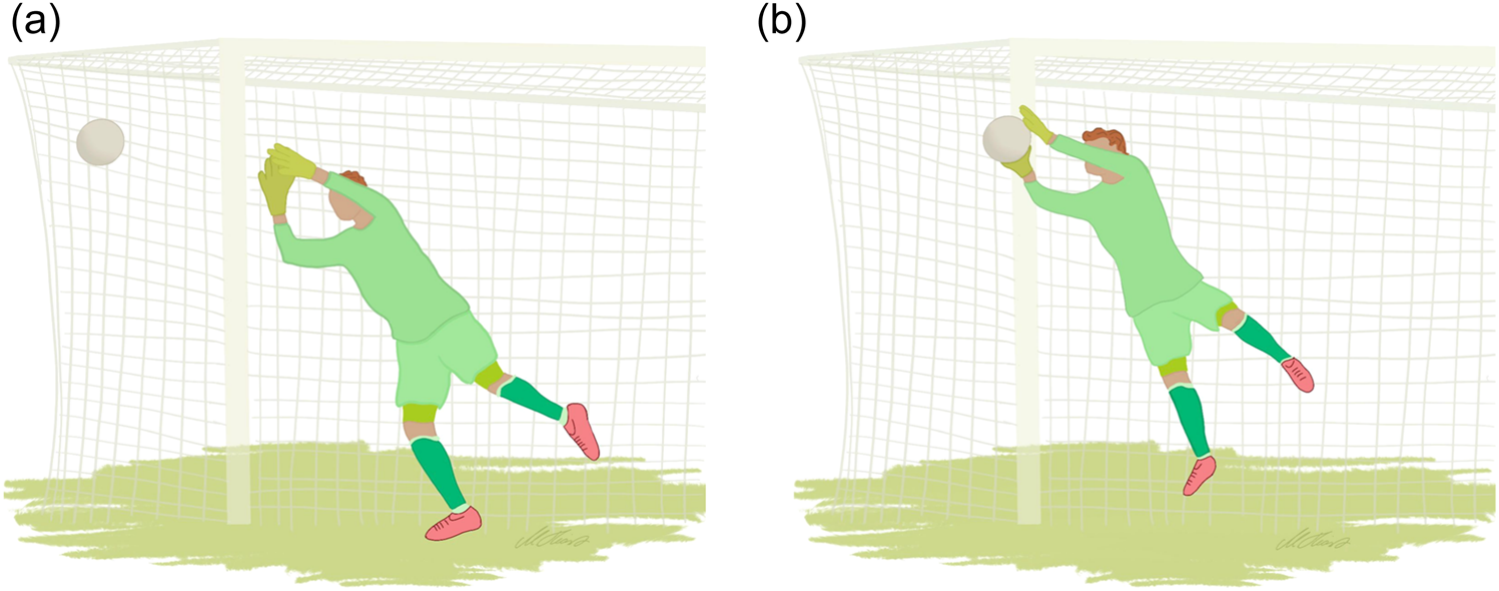
(a and b) Shoulder dislocation following saving the ball.

Two injury mechanisms were identified: a shoulder dislocation caused by a direct contact to the upper extremity by an opponent (Type 1; *n* = 22, 59%; Figure 1a,b) and a shoulder dislocation caused by catching a fall (Type 2; *n* = 14, 38%; Figure 2a,b). One additional case involved a goalkeeper dislocating his shoulder while saving the ball (*n* = 1, 3%; Figure 3a,b).

Players with a Type 1 mechanism had a mean age of 24 years (range, 18–26) and a median layoff time of 94 days (IQR, 67–118). In comparison, players with a Type 2 mechanism had a mean age of 26 years (range, 21–33; *p* = 0.026) and a median layoff time of 56 days (IQR, 20–104; n.s.).

In eight cases (22%), a heading situation was involved, equally distributed between Type 1 and Type 2 mechanisms (n.s.; Table 1). Other factors, including minute played (n.s.), player's position (n.s.), season half (n.s.), home versus away grounds (n.s.) and necessity of surgical intervention (n.s.), were similar between Type 1 and Type 2 mechanisms.

**Table 1:** Injury mechanisms and general characteristics.

		Type 1	Type 2
	Overall, *n* (%)	Direct contact, *n* (%)	Catching a fall, *n* (%)
Injury mechanisms			
Overall	37 (100)	14 (38)	22 (59)
Heading involved	8 (22)	3 (21)	5 (23)
Position			
Goalkeeper	1 (3)	0 (‐)	0 (‐)
Defender	13 (35)	4 (27)	9 (41)
Midfielder	14 (38)	5 (36)	9 (41)
Striker	9 (24)	5 (36)	4 (18)
Season			
First half	18 (49)	6 (43)	12 (55)
Second half	19 (51)	8 (36)	10 (45)
Pitch			
Home	20 (54)	8 (57)	11 (50)
Away	17 (46)	6 (43)	11 (50)
Surgery			
Yes	14 (38)	3 (21)	10 (45)
No	23 (62)	11 (79)	12 (55)

### Situational patterns

Type 1 and Type 2 mechanisms showed statistically significant differences in the player's action (*p* < 0.001) and type of contact (*p* = 0.005). However, player's speed (n.s.), direction of movement (n.s.), football‐specific action (n.s.), ball possession (n.s.) and pitch location (n.s.) were similar (Table 2).

**Table 2:** Situational patterns.

		Type 1	Type 2
	Overall, *n* (%)	Direct contact, *n* (%)	Catching a fall, *n* (%)
Player's action			
Running	5 (14)	5 (36)	‐
Sprinting	3 (8)	3 (21)	‐
Starting a sprint	1 (3)	1 (7)	‐
Stopping a sprint	2 (5)	2 (14)	‐
Change of direction	1 (3)	1 (7)	‐
Falling	15 (41)	‐	14 (64)
Landing	5 (13)	‐	5 (23)
Sliding	3 (8)	1 (7)	3 (14)
In the air	2 (5)	1 (7)	‐
Player's speed			
Fast	23 (62)	7 (50)	6 (27)
Medium	13 (35)	7 (50)	16 (73)
Slow	1 (3)	‐	‐
Direction of movement			
Forward	27 (73)	11 (79)	15 (68)
Backwards	1 (3)	‐	1 (5)
Sidewards	6 (16)	2 (14)	3 (14)
Downwards	2 (5)	‐	2 (9)
Down‐backwards	1 (3)	1 (7)	1 (5)
Football‐specific situation			
Heading	4 (11)	2 (14)	2 (9)
Tackling	7 (19)	5 (36)	2 (9)
Sliding tackle	4 (11)	1 (7)	3 (14)
Shooting	1 (3)	1 (7)	‐
Pressing	1 (3)	‐	1 (5)
Shielding the ball	3 (8)	3 (21)	‐
Saving the ball	1 (3)	‐	‐
Bicycle kick	1 (3)	‐	1 (5)
Hit during sprint duel	1 (3)	‐	1 (5)
Fall after tackling	7 (19)	‐	7 (32)
Fall after heading duel	2 (5)	1 (7)	1 (5)
Fall after dribbling	2 (5)	1 (7)	1 (5)
Fall after shielding the ball	1 (3)	‐	1 (5)
Landing after heading	1 (3)	‐	1 (5)
Ball receiving	1 (3)	‐	1 (5)
Type of contact			
Direct	19 (51)	14 (100)	5 (23)
Shoulder	9 (24)	8 (57)	1 (5)
Arm	8 (22)	4 (29)	4 (18)
Both	2 (5)	2 (14)	‐
Indirect	14 (38)	‐	17 (77)
Head	1 (3)	‐	1 (5)
Chest	2 (5)	‐	2 (9)
Hip	1 (3)	‐	1 (5)
Leg	3 (14)	‐	3 (14)
Foot	7 (19)	‐	7 (32)
None	4 (11)	‐	3 (14)
Ball possessing team			
Injured player	22 (59)	7 (50)	15 (68)
Injured player's teammate	1 (3)	1 (7)	‐
Opponent	13 (35)	5 (36)	7 (32)
No one	1 (3)	1 (7)	‐
Location on the pitch			
Own half	13 (35)	6 (43)	6 (27)
Opposite half	24 (65)	8 (57)	16 (72)
On‐field treatment			
Reposition attempt			
Yes	15 (41)	8 (57)	7 (32)
*Of that successful*	10 (67)	5 (63)	5 (71)
By himself	3 (20)	1 (13)	2 (29)
*Of that successful*	3 (100)	1 (100)	2 (100)
By team physician	11 (73)	6 (75)	5 (71)
*of that successful*	7 (64)	4 (67)	3 (60)
By teammate	1 (7)	1 (13)	‐
*Of that successful*	‐	‐	‐
No	15 (41)	3 (21)	11 (50)
Unclear	7 (19)	3 (21)	4 (18)
Reposition technique			
Traction‐countertraction	6 (40)	3 (38)	3 (43)
*Of that successful*	4 (67)	2 (67)	2 (67)
Milch	2 (13)	2 (25)	‐
*Of that successful*	2 (100)	2 (100)	‐
Spaso	2 (13)	2 (25)	‐
*Of that successful*	1 (50)	‐	‐
Self‐reduction	3 (20)	1 (13)	2 (29)
*Of that successful*	3 (100)	1 (100)	2 (100)
Unclear	2 (13)	‐	2 (29)
*Of that successful*	1 (50)	‐	1 (50)
Referee's decision			
Foul by the injured player	3 (8)	1 (7)	2 (9)
Foul by the opponent	8 (22)	2 (14)	6 (27)
No foul	26 (70)	11 (79)	14 (64)
Card			
Yellow card to the injured player	‐	‐	‐
Yellow card to the opponent	3 (8)	1 (7)	2 (9)
Red card	‐	‐	‐
No card	34 (92)	‐	‐

**Figure 4:**
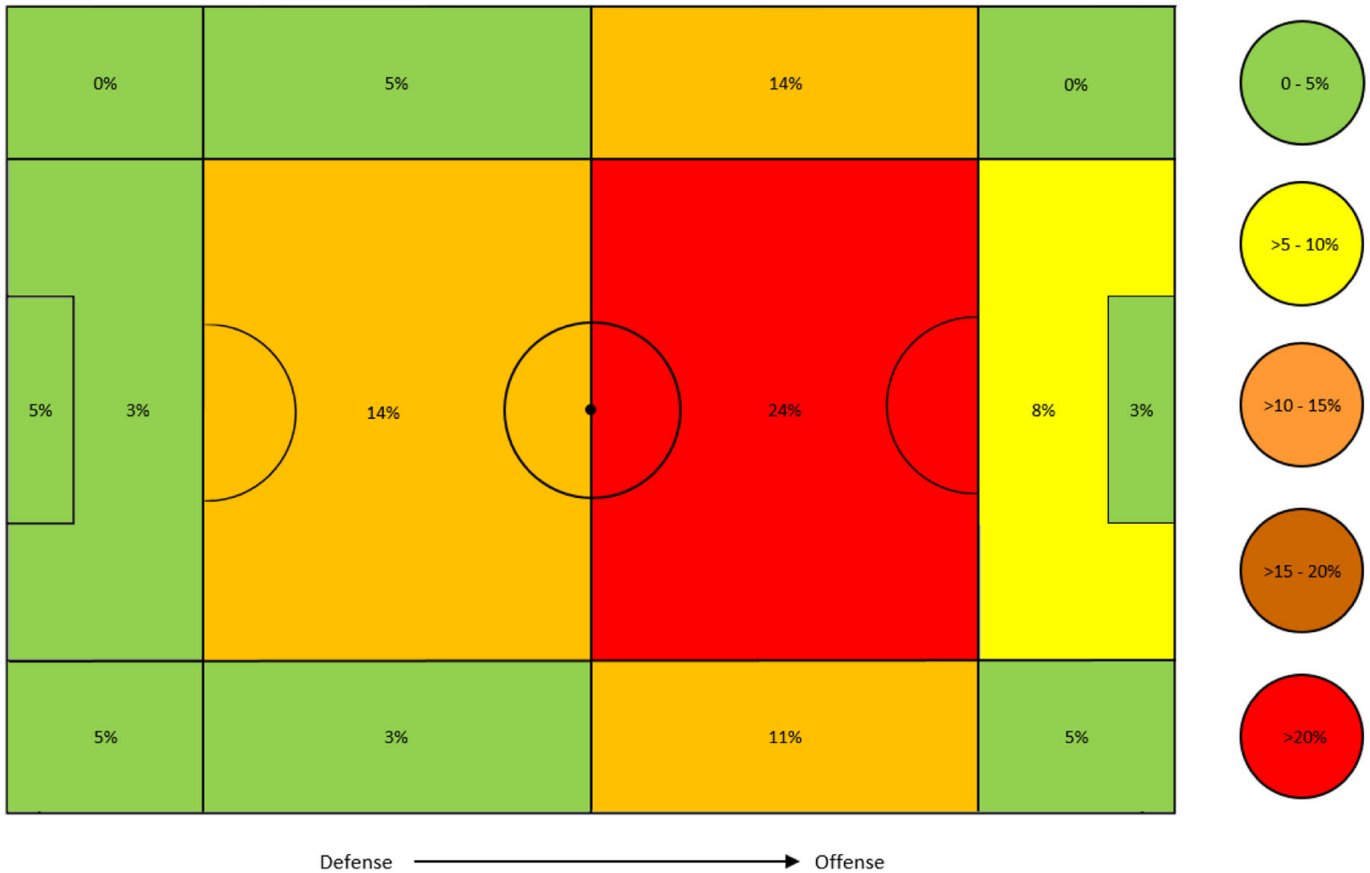
Overall shoulder dislocations (in percentage) by pitch location—graphic modified according to VBG‐Sportreport 2024 [32].

Overall, shoulder dislocations predominantly occurred in the offensive midfield and hardly in the areas around the corners and within the penalty area (Figure 4).

On‐field treatment varied but showed no statistically significant differences in reposition attempts (n.s.) and applied reduction techniques (n.s.). A successful on‐pitch reposition resulted in a median layoff time of 87 days (IQR, 45–104), while unsuccessful on‐pitch attempts had a median layoff time of 99 days (IQR, 15–111; n.s.).

Referee decisions regarding fouls (n.s.) and cards (n.s.) were also similar between both types of shoulder dislocation.

### Kinematic patterns

Trunk rotation (n.s.), shoulder joint version (n.s.), elevation n.s.), rotation (n.s.), elbow joint position in pronation/supination (n.s.) and flexion/extension (n.s.), as well as wrist joint position in extension/flexion (n.s.) at the moment of shoulder dislocation were also similar among both mechanisms of shoulder dislocation (Table 3).

**Table 3:** Kinematic patterns.

		Type 1	Type 2
	Overall, *n* (%)	Direct contact, *n* (%)	Catching a fall, *n* (%)
Rotation of the trunk			
To injured side	8 (22)	2 (14)	6 (27)
To uninjured side	‐	‐	‐
No rotation	29 (78)	12 (86)	16 (73)
Position of the shoulder joint			
Anteversion/retroversion			
Anteversion <90°	13 (35)	5 (36)	8 (36)
Anteversion 90°	1 (3)	‐	‐
Anteversion >90°	6 (16)	4 (29)	2 (6)
Neutral	9 (24)	3 (21)	6 (27)
Retroversion	8 (22)	2 (14)	6 (27)
Abduction/adduction			
Abduction <90°	14 (38)	6 (43)	7 (32)
Abduction 90°	3 (8)	2 (14)	1 (5)
Abduction >90°	15 (41)	3 (21)	12 (55)
Neutral	‐	‐	‐
Adduction	5 (14)	3 (21)	2 (6)
Rotation			
Internal rotation	18 (49)	6 (43)	11 (50)
External rotation	‐	‐	‐
Neutral position	13 (35)	5 (36)	8 (36)
Unclear	6 (16)	3 (21)	3 (14)
Position of the elbow joint			
Extension	1 (3)	‐	1 (5)
Neutral	1 (3)	‐	1 (5)
Flexion	35 (95)	14 (100)	20 (91)
Unclear	‐	‐	‐
Pronation	32 (6)	12 (86)	19 (86)
Neutral	‐	‐	‐
Supination	1 (3)	‐	1 (5)
Unclear	4 (11)	2 (14)	2 (9)
Position of the wrist			
Extension	13 (35)	2 (14)	10 (45)
Neutral	12 (32)	5 (36)	7 (32)
Flexion	1 (3)	1 (17)	‐
Unclear	11 (30)	6 (43)	5 (23)

## DISCUSSION

The main findings of our study are: (1) Shoulder dislocations in professional football are caused almost exclusively by direct contact with the upper extremity or due to catching a fall. (2) They regularly follow match situations that are hardly rated as a foul and subsequent medical on‐pitch treatment varies. (3) Kinematically, both types exhibit similar patterns in trunk rotation, as well as shoulder, elbow and wrist joint positions.

Shoulder dislocations in professional football are caused by direct contact with the upper extremity (Type 1) or due to catching a fall (Type 2). Football is considered a contact but not a collision sport, as such, the percentage of shoulder dislocations caused by a direct contact (38%) is non‐surprisingly lower compared to those of collision sports like American football or rugby, where 85% and 67% of shoulder dislocations are caused by a direct contact, respectively [3, 21]. Notable is the regular involvement of heading situations which are equally present in around one‐sixth of both types of shoulder dislocations and which have previously been identified as an important risk factor for injuries in football [1]. Both findings, the two distinct injury mechanisms and the regular involvement of heading situations, offer the possibility to implement targeted preventive measures like in‐air collision or fall training [4, 20].

In recent years, the FIFA11+ program, a comprehensive set of exercises, has been developed in order to reduce the injury burden in football. Interestingly, within the FIFA11+ program, only one exercise is described as a preventive measure for upper extremity injuries. Consequently, it appears that the severity of this injury is gravely underrepresented and its increasing incidence and considerable layoff times in professionals are neglected [18, 25, 26]. This is particularly frustrating because a tailored FIFA11+ program for goalkeepers has already been shown to effectively reduce upper extremity injuries—but more than 90% of shoulder dislocations occur in field players rather than goalkeepers [4, 26]. Preventive measures to address Type 1 mechanisms could include training drills focused on controlled ground and in‐air contact and impact management. This could include exercises designed to improve body positioning and protecting techniques during both intentional and incidental collisions, helping players stabilize their shoulders and upper extremities upon impact. Such training may also include reactive drills that simulate high‐contact scenarios, allowing athletes to practice safe collision responses and reduce injury risk. For Type 2 mechanisms, preventive measures could emphasize on specific fall‐training techniques aimed at minimizing shoulder impact. These could include rolling and landing drills designed to teach players how to disperse force and avoid extending their arms in a way that increases shoulder vulnerability. Practising controlled descent techniques, such as shoulder rolls and proper landing positions, could improve an athlete's ability to instinctively protect their shoulders during a fall. Notably, over the 10‐year observational period, no player experienced a subsequent dislocation to the opposite shoulder joint, emphasizing the traumatic aetiology of the injury and downplaying the role of potential non‐traumatic, constitutional factors for shoulder dislocation [22].

Interestingly, only one‐third of shoulder dislocations in professional football occur in situations rated as fouls by referees, suggesting that these injuries often result from routine match situations rather than hard tackles. Similarly, only one third of concussions arise from fouls, though yellow cards are awarded twice as often for concussions compared to shoulder dislocations (17% vs. 8%) [10]. These findings align with research in professional handball, where Luig et al. observed that ‘contact—but no foul play—was the most common mechanism associated with moderate and severe injuries’, with fouls accounting for just one third of all injuries [19].

The medical on‐pitch treatment varied with some medical teams successfully performing a shoulder reduction on‐field, some being unsuccessful and other medical teams making no attempt to reduce the shoulder joint on the pitch [28]. However, previous studies have shown that a delay between injury and the initial reduction attempt reduces the likelihood of successful reduction [12]. Furthermore, routine pre‐reduction radiographic examination may not be necessary for patients in their second and third decade, as the risk of fractures associated with shoulder dislocations in this age group is low [23]. Furthermore, reduction techniques varied, with some teams using outdated types of traction‐countertraction techniques like the Hippocrates method which is associated with neurovascular complications [24]. In professional sports, there's a growing trend towards standardized on‐pitch treatment protocols, especially for head injuries and cardiac arrest [1, 16, 31]. These findings highlight the need for standardized procedures for shoulder dislocations, including training medical staff and defining preferred reduction techniques [5, 28]. Standardization could theoretically improve outcomes by ensuring consistent, evidence‐based care that might minimize complications and expedite recovery. Establishing such protocols could be an important next step to investigate potential benefits for player health and return‐to‐play timelines.

Compared to shoulder injuries overall, shoulder dislocations occur quite early in a match with a mean time played by the individual player of 35 min compared to the 60% of all shoulder injuries that have shown to occur during the second half of the match [18]. This indicates that the incidence of shoulder dislocations is not predominantly triggered by overuse or fatigue. This finding is contrary to shoulder dislocations in professional rugby, where 36% of all shoulder dislocations have shown to occur in the final quarter of Rugby union matches indicating a potential ‘sign that players fail to prepare themselves for contact situations as successfully in the later stages of the game’ [21]. Regarding kinematic patterns, both dislocation types did not show statistically significant differences. Instead, shoulder dislocations followed the typical dislocation patterns of direct contact or abduction, extension and external rotation of the arm [27, 30].

Several limitations need to be acknowledged. (1) This study is a retrospective video analysis of in‐match shoulder dislocations of the 1. and 2. German Bundesliga. Shoulder dislocations that occurred during training are not included and could potentially follow different injury mechanisms as in‐match shoulder dislocations. As such, conclusions drawn from this study should be interpreted with caution. (2) The exact moment of a shoulder dislocation as well as its kinematics are sometimes difficult to identify as the injury is regularly embedded in a rapid, often at first glance unclear match situation with other players blocking the view and effects of clothing. However, the available video graphic content was comprehensive, of high quality and offered various perspectives from different camera angles. (3) Only male professional football teams were included and as such, no conclusions can be drawn to shoulder dislocations in professional female football as injury mechanisms, situational patterns, and kinematics could potentially be different in female professional football. (4) Although the results highlight variability in on‐pitch treatment methods observed, we could not directly measure outcomes associated with specific treatment approaches. (5) The study lacks statistical power and is unable to perform statistical analyses on certain checklist categories due to insufficient case numbers. Nonetheless, it includes all 37 consecutive first‐time shoulder dislocations from the 1. and 2. German Bundesliga over a span of more than 10 years, making it the most comprehensive study of this specific injury in professional male football to date.

## CONCLUSION

Shoulder dislocations in professional football typically occur through direct contact or catching a fall, indicating a potential role for specific preventive measures.

## AUTHOR CONTRIBUTIONS

Kristian Nikolaus Schneider designed the study. Tim Schachtrup and Kristian Nikolaus Schneider collected and analysed the data. Kristian Nikolaus Schneider, Theodoros Zafeiris, Tim Schachtrup, Mirkka Lynn Hiort, Blanca Julie Degener and Christoph Theil were responsible for data management and preparation of figures. Kristian Nikolaus Schneider, Tim Schachtrup and Christoph Theil wrote the manuscript. Georg Gosheger helped with data analysis and editing the manuscript. All authors read and approved the final manuscript.

## CONFLICT OF INTEREST STATEMENT

The authors declare no conflicts of interest.

## ETHICS STATEMENT

The study was approved by the Ethikkommission der Ärztekammer Westfalen/Lippe (reference number 2023‐215‐f‐S) and performed in accordance with the Declaration of Helsinki. Patient consent was waived due to ethics committee approval. All figures and tables are exclusively original, there is no material from other sources. The authors affirm that the study's supporting data are contained within the article.

## Data Availability

The data that support the findings of this study are available on reasonable request from the corresponding author.
